# The built environment, purpose-specific walking behaviour and overweight: evidence from Wuhan metropolis in central China

**DOI:** 10.1186/s12942-024-00361-y

**Published:** 2024-01-25

**Authors:** Sanwei He, Shan Yu, Lina Ai, Jingya Dai, Calvin King Lam Chung

**Affiliations:** 1 School of Public Administration, Zhongnan University of Economics and Law, Wuhan, 430073 China; 2 Department of Land Economy, University of Cambridge, Cambridge, UK; 3 Department of Community, Culture and Global Studies, University of British Columbia Okanagan, Kelowna, Canada; 4 Department of Geography and Resource Management, The Chinese University of Hong Kong, Hong Kong, China

**Keywords:** Objective environment, Subjective environment, Purpose-specific walking, Health, Multi-group structural equation model

## Abstract

The impact of objective and subjective environmental factors on health outcomes has been a topic of significant debate, with a growing body of research acknowledging the role of a physically active lifestyle in promoting health. However, consensus regarding their precise influence remains elusive. This study contributes to these discussions by exploring how individual health outcomes correlate with transport and leisure walking behaviours, set against both the objective and subjective aspects of environmental influences in the context of Wuhan, an inland Chinese megacity. Street view images, multi-source geospatial data and a questionnaire survey were employed to characterise the “5D + Greenery” objective and perceived characteristics of the neighbourhood environment. Multi-group structural equation modelling was utilised to unravel the complex relationship and gender heterogeneity among environmental factors, purpose-specific walking, and overweight. Our results suggest that both objective land use diversity and perceived convenience are significantly associated with overweight. The accessibility of local service facilities and visible greenery promote both transport and leisure walking. While perceived neighbourhood safety encourages transport walking, perceived walkability is positively correlated with leisure walking. Notably, leisure walking, usually considered beneficial, presents a positive association with overweight conditions, acting as a mediation. Gender disparities exist in pathways between the environment and purpose-specific walking, as well as weight. The findings lend support to the planning of an activity-supporting built environment as a crucial strategy for obesity prevention.

## Introduction

Physical inactivity is widely recognised as a prevalent risk factor for non-communicable diseases, an obvious example of which is overweight [2]. The gravity of the situation is testified by the World Health Organization’s [71] launch of Global Action Plan on Physical Activity 2018–2030, which calls for a systemic response to promoting physical activities, underscoring the crucial role urban planning can play in developing an “active environment” that encourages physical activity, especially walking [17]. Walking encompasses both destination-specific and leisure walking for recreation, and understanding the diverse walking behaviours in relation to urban environments and health outcomes is essential for creating walk-friendly cities [36, 67, 69, 70].

Obesity is largely linked to an energy imbalance where energy expenditure is less than calorie intake, often resulting from sedentary lifestyles and high vehicle reliance [62]. In this context, as a form of physical activity easily incorporated into daily activities, walking can increase energy expenditure, promote better physical and mental health, and reduce obesity risk [8, 72]. Furthermore, regular walking is beneficial in enhancing physical fitness, decreasing depression, and psychological distress, ultimately acting as a protective factor against obesity and chronic conditions [29, 57]. Given the beneficial health outcomes of walking behaviours, numerous planning and public health studies have delved into the structural relationships between the built environment, walking, and health outcomes such as obesity [31]. For instance, pedestrian-friendly objective environments with high-density, mixed land-use, and well-designed connected sidewalks—characteristics of an active community environment as proposed by the United States Centers for Disease Control and Prevention—can encourage walking behaviours [11, 52]. These walking behaviours can then increase energy expenditure, reduce obesity, and thereby improve overall health [31].

However, the impact of walking behaviours on health outcomes can vary, depending on the purpose of walking, i.e., whether it is utilitarian or recreation [51, 60]. Utilitarian walking, or transport walking, generally goal-directed towards routine destinations, is less influenced by physical environments and more pre-determined,on the other hand, leisure walking, conducted for recreation or exercise, is spontaneous and flexible, highly sensitive to the quality of the built environment [33]. Considering these distinct motivations and attributes, it becomes crucial to consider utilitarian and leisure walking behaviours separately in understanding their mediating effects on health outcomes in relation to the built environment [61].

Studies on the relationship between the neighbourhood environment and physical activity focus mostly on the Global North. Different from low-density neighbourhoods in developed countries, high-rise and high-density residential neighbourhoods are mushrooming in Chinese cities to ease the pressure of urban land use, which might pose an adverse impact on residents’ health consequences. With an obese population second only to the United States, the worrying trend of obesity was acknowledged by “Healthy China 2030”, the Chinese government’s blueprint to address challenges in maintaining and promoting public health. Released in 2016, the blueprint emphasises that the idea of health should be integrated with people-oriented urban planning and community governance. It aims to raise the share of the Chinese population engaging in regular exercise to 40% by 2030. Against this background, implementing health-related planning interventions and strategies requires a detailed understanding of the varied impact of purpose-specific walking behaviour among different groups.

This study seeks to untangle the complex interplay between objective and subjective measures of the built environment, purpose-specific walking behaviour, and health outcomes, particularly within the context of Wuhan, an inland Chinese megacity. Extending the conventional “5D” framework, we include measures of eye-level street greenery in the objective neighbourhood environment and employ structural equation modelling (SEM) to reveal the nuanced pathways between these components [4, 12]. Importantly, we also examine gender disparities in these relationships, providing valuable insights into a critical yet under-researched context in developing countries like China.

This paper contributes to existing literature in the following aspects. First, we simultaneously consider the objective neighbourhood conditions derived from geospatial data and subjective neighbourhood characteristics reported by residents, which will be helpful to capture the varied impact on purpose-specific walking and adult overweight. Second, SEM is utilised to reveal the pathways between objective and subjective environmental measures, and overweight, as well as the potential mediations of leisure or transport walking. Third, gender differences in environmental perceptions and health-related outcomes are rarely examined in developing countries like China. Multi-group SEM is employed to investigate the gender-specific disparities in associations between objective and subjective environment, purpose-specific walking and overweight.

## Literature review

### The objective and subjective measures of built environment and purpose-specific walking

Advocates of active lifestyles often encompass walking as a cost-efficient daily physical activity. Understanding the impact of the built environment on walking behaviour necessitates differentiating between transport (or utilitarian) and leisure (or recreation) walking, as each is uniquely influenced by the environment and contributes differently to health outcomes [27, 34]. Previous empirical studies measuring objective environments based on classical “3D” or extended “5D” frameworks confirm that more compact, diverse, and pedestrian-oriented neighbourhoods or street configurations provide amenities for walking [4, 12, 28]. For instance, in low-density cities such as those in the United States and Australia, an increase in residential density promotes transport walking [9, 44], whereas a negative correlation occurs in a high-density city, Nanjing, China [75]. Focusing on women in socioeconomically disadvantaged neighbourhoods, Cleland et al. [6] found that density of destinations and connectivity are positively associated with transport walking and negatively associated with leisure walking. It is observed that transport walking has significantly positive associations with GIS measure of accessibility but not perceived measure of accessibility to amenities in Singapore [43]. Empirical evidence from Sendai, Japan showed that people living in neighbourhoods with greater overall green visibility spent more time per week leisure walking [53]. However, these relationships are not uniform and can vary considerably across different urban settings.

Further complexity is added when considering subjective measurements of the built environment, encapsulating individual perceptions and experiences [77]. Residents' perceived “practical” features are important for transport walking, while ‘enjoyment’ features are more closely related to walking for pleasure [7, 59]. Su et al. [59] found high-quality precepted aesthetic were associated with more time in leisure-time physical activities in women in Hangzhou. Evidence from Taiwan showed that perceived land-use mix diversity and pedestrian safety were associated with purposive or transport-related walking, while aesthetics and crime safety were associated with leisure walking [20]. A survey about mothers’ local environmental perceptions in Melbourne found that perceived public transport accessibility and trusting neighbours were predictive of increases in walking for leisure, while perceived connectivity and local traffic speed increased transport-related walking [7]. Further nuanced research is needed to unpack the complex interplay between the objectively and subjectively measured built environment, and purpose-specific walking. [35, 70]

### Importance and impact of gender differences

It is critical to understand how these gender-specific interactions might influence health interventions and strategies [47, 74]. Men and women engage differently in walking for transport and leisure, which can lead to varying health outcomes [16, 32]. For example, women’s daily lives are occupied by more varied obligations, including employment, household work and caregiving, which is largely due to traditional social norms and gender roles [55]. More exposed to their neighbourhood, women ‘s physical activities were found to be more sensitive to their effects and to make different behavioral choices [47, 49].

Furthermore, gender might interact with the built environment to influence walking behavior. Perceived safety is the key factor that distinguishes men's and women's walking choices [50]. Women were significantly less likely to undertake any walking in unsafe environments, while men's walking is unaffected by these concerns [13]. The results, however, are inclusive but inconclusive as environmental characterization encompasses objective and subjective measurements, and the purposes of walking are distinguished. Adlakha and Parra [1] observed that transport-related physical activity was dominant among women, with less activity during leisure time. It was found that density of destinations and connectivity are positively associated with transport walking and negatively associated with leisure walking for women in socioeconomically disadvantaged neighborhoods [6]. Gao et al. [15] found that female residents who lived in neighborhoods with higher road sky view index and increased intersection density showed lower risk of increased BMI. In conclusion, the influence of gender on the relationships between the built environment, walking behaviors, and health outcomes necessitates further research. A nuanced understanding of these gender-specific interactions could help in developing more effective health-promotion strategies.

### Conceptual framework

**Fig. 1:**
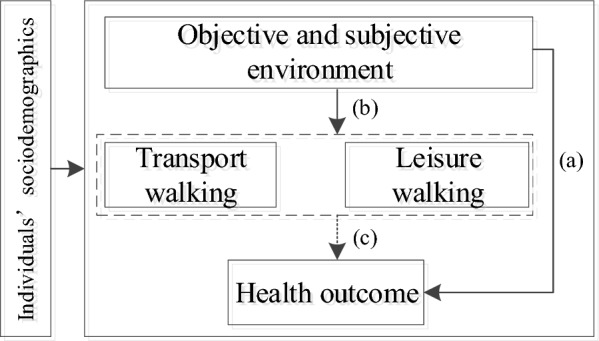
The conceptual framework

This study proposes a comprehensive conceptual framework (Fig. 1) to investigate the multifaceted effects of the built environment (objective and subjective) on purpose-specific walking behaviour and health outcomes. The framework facilitates a better understanding of the complex relationships between these variables and addresses the research gaps identified in the previous subsections.

The first element of the framework investigates the direct effect of the built environment, both objectively and subjectively measured, on purpose-specific walking behaviours and health outcomes. By focusing on the direct effect, we can better understand the immediate impact of the built environment on walking behaviours and health (marked by path (a) and path (b)).

Next, the framework examines the mediating role of walking behaviours (both transport and leisure) in the relationship between the built environment and health outcomes. This element is crucial because walking behaviours can mediate the impact of the built environment on health outcomes [marked by path (c)].

## Data and methods

### Study area

**Fig. 2:**
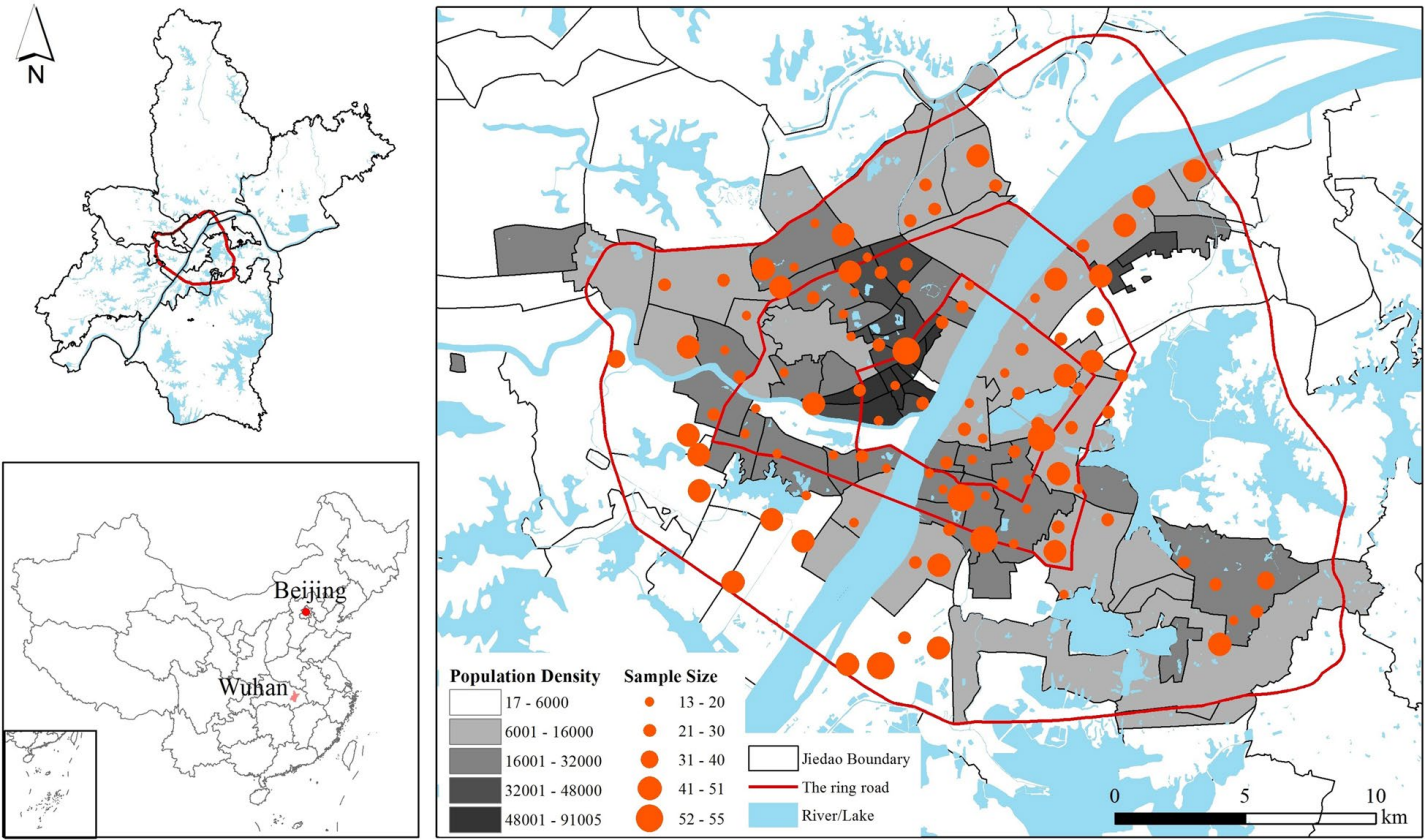
Study area and sampled neighborhoods (Population density, in persons/km^2^, calculated according to China’s Sixth National Population Census in 2010)

In this study, we focus on the case of Wuhan, a fast-growing Chinese megacity along the Yangtze River (Fig. 2). With an economy dominated by tertiary (62.5% of local GDP) and secondary (35.0% of local GDP) sectors [73], Wuhan boasts one of the highest GDP growth rates in China. It spans 8569.15 km^2^ as an administrative unit and is home to 13.65 million people. Before making headlines the early epicentre of the COVID-19 pandemic, Wuhan was known for its official goal of becoming China’s model healthy city. In its “Healthy Wuhan 2035 Plan”, announced in 2018, the local government seeks not only to improve the city’s medical infrastructure—a goal that, in hindsight, should have been achieved earlier—but also to introduce preventative interventions to promote the fitness of local residents. For the latter, one of the official targets is to raise the local population with regular exercise by over four million by 2035. Against this backdrop, this study is interested in how well Wuhan’s neighbourhood environment can contribute to this public health goal through its influence on walking as a form of daily physical exercise.

In our study, neighbourhoods are defined according to the concept of residential communities, also known as ‘‘*xiaoqu*’’ in the Chinese context. These residential communities, or ‘‘*xiaoqu*’’, represent typical forms of residential organization in urban China, evolving from work units to more diversified forms post the Chinese economic reform. Characteristics such as controlled entrances, safety, and available services, differentiate these communities, thereby influencing residents' perceptions and experiences of their immediate environment.

### Data sources and data description

#### Data sources

Our study builds upon a questionnaire survey of subjective perceptions of neighbourhood environment, walking habits, health outcomes and individuals’ socioeconomic attributes in Wuhan conducted in November 2019. Adopting stratified random sampling, we identified 106 neighbourhoods from 54 *jiedao*^1^ units in the inner part of Wuhan. The number of neighbourhoods sampled in each *jiedao* of Wuhan is proportional to the population of such *jiedao*. In each neighbourhood, the number of households sampled is proportional to the size of the neighbourhood: 15 for neighbourhoods with 750 or fewer households, 25 for those with 750–1450 households, and 50 for those with over 1450 households. Altogether 2385 valid responses were received.

The questionnaire consists of three parts. The first part gauged respondents’ perception of their neighbourhood environment based on their likelihood of agreeing with five statements on a scale of 1 (extremely unlikely) to 4 (extremely likely). The statements are structured around five aspects of the subjective environment, i.e., convenience, walkability, security, aesthetic appeal and residential crowdedness (see Sect. "Data description" for details). The second part measured respondents’ walking habits in terms of their aggregate duration of walking for transport and leisure in the week before they participated in the survey. The final part collected respondents’ demographic and socioeconomic information, as well as—for BMI calculation—their weight and height measurements.

The survey is complemented using a variety of georeferenced data to investigate objective measurements of the environment of the sampled neighbourhoods. First, georeferenced data on street networks and points of interest (POIs) were extracted from Baidu Maps (https://map.baidu.com/). Following the official statistical classification of socioeconomic activities in China, we categorised the POIs into nine functional classes: financial service, research and education, culture, health service, leisure and recreation, transport, government agencies, catering, and lodging. Second, 1622 roads were identified in the study area, and 12,319 location points were generated along the road networks at an interval of 100 m. The geographic coordinates of these locations were used to request Baidu Street View images through the application programming interface (API). We collected 33,516 images giving a 360  view of different parts of the streetscape in the study area in 2021. Although there is a potential time lag, the street-level greenery we focus on typically includes trees and larger greenery installations that do not fluctuate dramatically within a short time span. Then a deep learning model and image semantic segmentation technique were utilised to evaluate the greenery coverage at each location point. Third, in the absence of official demographic data at the neighbourhood scale, the WorldPop dataset with a 100-m resolution was drawn upon to estimate neighbourhood population density.

#### Data description

(1) Health outcome measured by overweight

BMI is used as a proxy of respondents’ health outcomes, particularly in physical terms (i.e., weight [in kg]/square of height [in m]). Although BMI does not necessarily reflect uniform body fat distribution or intergroup “fatness” disparity, it is a practical and internationally acknowledged indicator of overweight. As a measure of body fat based on height and weight, it can be utilised to filter respondents with potential health problems based on weight categories. Following the suggestion of China’s National Health and Family Planning Commission (NHFPC), we categorise respondents with a BMI of 24 or above as being overweight.

(2) Objective environment

Incipient research assumes that residents who live in the same neighbourhood are exposed to the same physical environment that conditions their physical activities [77]. This assumption gives rise to a focus on assessing the neighbourhood environment in objective terms. Many of these assessments are structured around a “5D” framework to quantify the effects of the built environment on physical activities: density (e.g., population density or employment density), diversity (e.g., land-use mix), design (e.g., the percentage of road intersections or road connectivity), destination accessibility (e.g., access to amenities) and distance to transit (e.g., distance to metro stations) [12, 18, 28, 68]. Inspired by Sarkar et al. [54], we argue that objective greenness should also be counted as a walking motivator and a positive factor for physical health.

(3) Subjective environment

Shaped by the diversity of personal experiences and socioeconomic status, individuals may perceive the same neighbourhood environment differently [77], leading to heterogeneous walking motivations. Generalising from the literature [24, 26, 48, 54], we identify five key aspects of subjective environmental perceptions: convenience (the neighbourhood’s accessibility to various facilities), walkability (the ease of walking within a neighbourhood), security (safety and crime-freeness), aesthetic appeal (the aesthetic value of the neighbourhood’s physical setting) and residential crowdedness (perception of spatial limitation in the neighbourhood [58]. The estimated geographic context is the resident's daily activity circle, which is an area accessible within a 15 min walk from their home, effectively covering the services available nearby their neighbourhood.

(4) Purpose-specific walking behaviour

Walking behaviour was assessed based on the duration and frequency that individuals engaged in transport/leisure walking in a week. For leisure walking, two questions were asked: “During the last 7 days, how many days did you walk for at least 10 min at a time for fun, relaxation, exercise, or to walk the dog?” and “How much time did you usually spend walking on one of those days?”. For transport walking, another two questions were asked: “During the last 7 days, how many days did you walk for at least 10 min at a time for education, commuting, shopping, medical service, eating/drinking, changing mode and others?” and “How much time did you usually spend walking on one of those days?”. Thus the continuous scores for leisure/transport walking were calculated with the following formula: minutes of activity per day × days per week. In addition, the measurement of walking is tailored to the geographic context of a resident's daily activity circle, which is aligned with the area of subjective environment measurement.

(5) Individuals’ socioeconomic attributes

The relationship between the built environment and weight should not be analysed irrespective of the residential self-selection effect because people may select a particular neighbourhood according to their travel attitudes and socioeconomic conditions [19]. Taking into account the residential self-selection effect, we suggest the following socioeconomic indicators as key confounders to be considered in the model: gender, age, marital status, household size, car ownership and homeownership.

### Methods

#### The “5D + Greenery” framework of the objective environment

Adapting from the ‘5D’ framework, we use six variables to quantify characteristics of the built environment within a 15 min walking distance, operationalized as a 1-km road network buffer, around each neighbourhood:

(i) Population density: ${den}_{i}={pop}_{i}/{A}_{i}$, where ${pop}_{i}$ denotes the total number of residents in the ${i}^{th}$ neighbourhood, and ${A}_{i}$ is the area of the buffered neighbourhood.
(ii) Diversity of land use: ${div}_{i} =-{\sum }_{m=1}^{k}\,{poi}_{mi}\,\,ln({poi}_{mi})/lnk$, where $k$ is the categories of POI types (nine for this study), and ${poi}_{mi}$ is the percentage of the ${m}^{th}$ POI category to the total number of all POIs in the ${i}^{th}$ neighbourhood.
(iii) Design of road network: ${des}_{i}$, the number of road intersections in the ${i}^{th}$ neighbourhood.
(iv) Destination accessibility: ${acces}_{i}$, the number of service facilities (e.g., catering, banks, leisure and entertainment) in the ${i}^{th}$ neighbourhood.
(v) Distance to transit: following Ye and Titheridge [76], we adopted the number of bus stops (a common mode of public transit in Wuhan) as the alternative measurement of ${tran}_{i}$ in the ${i}^{th}$ neighbourhood.
(vi) Greenery: ${green}_{i}$, the level of the human-viewed street greenery (measured by green view ratio) of the ${i}^{th}$ neighbourhood.

#### Deep learning and image semantic segmentation to evaluate viewed greenery

Green spaces were quantified through three steps: data acquisition, model training, and result analysis. First, street view images were obtained through the Baidu Maps API at sampling points to capture 360  panoramic pictures, which better reflect pedestrian perception of urban greenery than overhead-view GIS measures or four-directional street view images. Second, the DeepLab V3 semantic segmentation model was used to identify green elements. The model was generated after deep learning of 1,000 manually labelled street view pictures based on convolutional neural networks. Third, the ratio of greenery pixels to the total pixels was used to assess the level of viewed street greenery, and the average value of all points within the neighbourhood was used to evaluate the level of human-viewed greenery.

#### Structural equation modelling for unravelling the mechanisms of the neighbourhood environment on health

SEM was performed to unravel the intricate mechanisms among variables of subjective and objective neighbourhood environment, walking behaviour and health outcomes. Based on the conceptual framework (Fig. 1), we set the objectively and subjectively measured environmental factors and socioeconomic variables as exogenous observational variables, and walking duration and overweight as endogenous observational variables. We took the following steps to construct an appropriate structural equation model. First, validity and reliability tests were conducted to ensure that the questionnaire survey about walking behaviour and environmental perception provides valid and reliable information. Second, the model was constructed on the basis of the conceptual framework, and confirmatory factor analysis was conducted to identify the insignificant paths. Paths without significant impact were deleted to optimise the model. Third, the maximum likelihood method, a popular and effective method to estimate the parameters of the structural equation model, was applied. Since the survey data in the same neighbourhood may be spatially correlated, the clustering standard error instead of the traditional common standard error is used to estimate the model, thereby avoiding the amplification of the effect of neighbourhood environmental variables. Fourth, statistics such as Akaike Information Criterion (AIC) and Bayesian Information Criterion (BIC) were used to assess the goodness of model fit. To investigate whether there are group differences in the influence mechanism of neighbourhood environment on walking and overweight, we performed t-tests to compare the differences between the male and female groups. Furthermore, multi-group SEM was adopted to further analyse the gender differences in the influencing mechanisms. The AMOS software was used for this analysis.

## Results

### Descriptive statistics

Table 1 describes the summary and t-test statistics of the sample. Overall, the average time that respondents spent on transport walking was 82.84 min, while that on leisure walking was 112.78 min. There are significant differences in walking time between the male and female groups; women tend to have more walking time both for transport and leisure than men. And the high standard deviation for both categories indicate notable variation in walking time. The mean value of the respondents’ BMI is 22.19, which is within the healthy range, while overweight respondents (BMI of 24 or above) made up a quarter of the sample. BMI and overweight indicators also showed significant gender differences. On average, the male group tended to have a higher BMI (mean = 23.05) than the female group (mean = 21.41). The proportion of male respondents who were overweight reached 34%, twice as high as female respondents.

**Table 1:** Descriptive statistics

Variables	Description	Male	Female	Overall
		Mean/% (SD)	Mean/% (SD)	Mean/% (SD)
Individual-level variables				
Gender	1 (Male); 0 (Female)	N.A	N.A	0.48 (0.5)
Age	Age of respondents	39.02 (0.35)	40.03 (0.35)	39.54 (0.25)
Marital status	1 (Married and living with spouse) 0 (Single, divorced or widowed)	74.76 25.24	81.87 18.13	78.46 21.54
Household size	Number of family members living together	3.7 (1.19)	3.83 (1.22)	3.77 (1.2)
Car ownership	1 (One family owing at least one car) 0 (Having no car)	68.44 31.56	67.27 32.73	67.83 32.17
Homeownership	1 (Owning a home) 0 (Renting or others)	78.67 21.33	76.27 23.73	77.52 22.48
Objective environment				
Density	Population density within 1 km-buffered neighbourhood (persons/ha)	1.77 (0.79)	1.76 (0.79)	1.77 (0.79)
Diversity	Intensity of land use mix, measured on a scale from 0 (least diversified) to 1 (most diversified)	0.74 (0.06)	0.75 (0.07)	0.74 (0.06)
Design	Number of road intersections in the neighbourhood	3.59 (1.29)	3.52 (1.33)	3.56 (1.32)
Access	Number of service facilities in the neighbourhood	1.04 (0.85)	0.99 (0.85)	1.01 (0.85)
Transit	Number of bus stops in the neighbourhood	0.15 (0.07)	0.15 (0.07)	0.15 (0.07)
Green	The human-viewed street greenery	0.25 (0.07)^*^	0.24 (0.07)^*^	0.25 (0.07)
Subjective environment				
Convenience	My neighbourhood is convenient to various facilities	3.49 (0.97)^*^	3.42(0.97)^*^	3.45 (0.97)
Walkability	My neighbourhood is friendly to walk	2.64 (0.65)	2.64(0.61)	2.64 (0.63)
Aesthetics	My neighbourhood is aesthetically appealing	2.64 (0.68)^*^	2.69(0.62)^*^	2.67 (0.65)
Safety	My neighbourhood is safe and crime-free	2.83 (0.59)	2.81 (0.6)	2.82 (0.6)
Crowd	My neighbourhood is densely populated	2.85 (0.59)	2.84 (0.58)	2.84 (0.59)
Walking behaviour				
Transport walking	Time spent on transport walking (e.g. commuting or shopping) per week (minutes)	74.79 (107.9)^***^	90.27 (93.91)^***^	82.84 (101.14)
Leisure walking	Time spent on leisure walking (e.g. for park visit or recreation) per week (minutes)	106.88 (132.82)^**^	118.21 (129.64)^**^	112.78 (131.27)
Health outcomes				
BMI	Weight (kg)/square of height (m)	23.05 (2.63)^**^	21.41 (2.94)^***^	22.19 (2.91)
Overweight	1 (BMI ≥ 24); 0 (0 < BMI < 24)	0.34 (0.47)^***^	0.17 (0.38)^***^	0.25 (0.44)

*N.A.* not applicable. Each of the five subjective environment variables was measured by respondents’ agreement with a given statement on a scale from 1 (extremely unlikely) to 4 (extremely likely)

^*^p < 0.1

^**^p < 0.05

^***^p < 0.01

### Structural equation modelling

Table 2 presents our SEM-based analysis of the association of walking behaviour and overweight with a range of attributes of the respondents’ socioeconomic status, their objective and their subjective neighbourhood environment, respectively. Prior to conducting the analysis, we tested for multicollinearity and the Variance Inflation Factors (VIFs) were below 3. Our model passed the sensitivity checks with a root mean square error of approximation (RMSEA) of 0.021 (below the 0.06 threshold) and comparative fit index (CFI) of 0.997 (above the 0.90 threshold) [21, 37].

**Table 2:** Standardized direct effects of the built environment on walking behavior and overweight

Variables	Transport walking		Leisure walking		Overweight	
	Coef.	S.E	Coef.	S.E	Coef.	S.E
Individual-level variables						
Gender	− 0.083^c^	4.130	− 0.041^b^	5.279	0.205^c^	0.017
Age	0.093^c^	0.220	0.147^c^	0.281	0.113^c^	0.001
Marital status	− 0.069^b^	6.616	− 0.024	8.458	0.08^c^	0.027
Household size	0.022	1.782	0.069^c^	2.278	0.059^c^	0.007
Car ownership	− 0.040^a^	4.840	0.007	6.187	− 0.010	0.020
Homeownership	0.038	5.749	0.046^a^	7.350	0.033	0.024
Objective environment						
Density	− 0.071^b^	0.036	− 0.014	0.046	0.003	0.001
Diversity	0.026	35.786	0.034	45.747	0.037^a^	0.146
Design	0.032	0.024	− 0.049	0.031	− 0.013	0.001
Access	0.111^c^	0.036	0.105^c^	0.046	− 0.010	0.001
Transit	− 0.022	0.345	− 0.095^c^	0.441	− 0.036^b^	0.001
Green	0.091^c^	0.340	0.112^c^	0.434	− 0.003	0.001
Subjective environment						
Convenience	− 0.022	2.245	0.018	2.870	0.209^c^	0.009
Walkability	0.008	3.843	0.053^b^	4.913	0.016	0.016
Aesthetics	− 0.012	3.717	0.011	4.752	− 0.031	0.015
Safety	0.054^c^	3.917	0.026	5.008	− 0.023	0.016
Crowd	0.033^a^	3.625	0.034^a^	4.634	− 0.012	0.015
Walking behaviour						
Transport walking					− 0.001	0.001
Leisure walking					0.026^a^	0.001

Model performance: CMIN/DF 2.01; GFI 0.998; CFI 0.997; RMSEA 0.021; IFI 0.997; AGFI 0.982; NFI 0.994; AIC 420.241

^a^10% significance level; ^b^5% significance level; ^c^1% significance level

For individual-level variables, gender, age and marital status matter for transport and leisure walking. Women, unmarried and older people were more likely to walk for transport purposes. A larger household encouraged leisure walking. As for association with one’s BMI, men, elderly, married individuals, homeowners and members of larger households were more likely to have a higher BMI.

For objective environmental measures, many of them were significantly associated with walking behaviour. Population density (*density*) showed a negative effect on transport walking at the 5% significant level (− 0.071). Furthermore, both transport walking and leisure walking were also promoted by a higher exposure to local service facilities (*access*) and neighbourhood greenness (*green*). Land use diversity (*diversity*), which showed insignificant association with either type of walking, was significantly related to individual overweight in a positive way.

For subjective environmental measures, perceived neighbourhood crowdedness (*crowd*) is significantly positively associated with both transport walking and leisure walking. A walking-friendly environment (*walkability*) was significantly conducive to walking for leisure, while a safe and crime-free environment (*safety*) could significantly encourage transport walking. Neither a neighbourhood’s accessibility measurement (*convenience*) nor its aesthetic appeal (*aesthetics*) mattered the respondents’ walking behaviour. Moreover, perceived neighbourhood convenience to various facilities (*convenience*) was significantly and positively associated with individual overweight.

For the association between walking behaviour and overweight, we found that leisure walking was significantly associated with one’s overweight and, surprisingly, in a positive manner. This could be explained by the growing night-time economy in China. China’s night-time consumption reached 30 trillion RMB in 2020 and is expected to continue growing at a scale of approximately 17% per year [22] Wuhan has been shortlisted for the top ten cities in China’s night-time economy. People usually take a leisure walk after getting off work in the neighbourhood. A leisure walk along widespread snack streets or night markets tends to bring a high-calorie intake of snacks, street foods and desserts, which leads to a high possibility of gaining weight.

### Mediation analysis

We further considered whether and how the objective and subjective environment may indirectly affect individual’s health outcome via specific-purpose walking. Table 3 depicts the pathways through which the indirect effects of the environmental metrics were exerted via different behaviours. Since the mediating effect of traffic walking is not significant, we only present results for leisure walking. An objective environment with higher population density and more bus stops (*density*, *transit*) tends to reduce the likelihood of being overweight through leisure walking. However, accessibility to service facilities (*access*) and greenery coverage (*green*) may lead to a higher likelihood of overweight through the mediation of leisure walking. For subjective measurements, perceived walkability, safety and crowdedness demonstrate a positive indirect impact on overweight through leisure walking.

**Table 3:** Indirect effects of the built environment on overweight: walking behaviour as mediators

Indirect paths	Standardized coefficient	Bias-corrected 95% confidence interval		Percentile 95% confidence interval	
		Lower	Upper	Lower	Upper
Density → Leisure walking → Overweight	− 0.0004^a^	− 0.049	0.052	− 0.048	0.054
Diversity → Leisure walking → Overweight	0.0009^a^	− 0.003	0.076	− 0.001	0.078
Access → Leisure walking → Overweight	0.0028^a^	− 0.065	0.050	− 0.063	0.051
Transit → Leisure walking → Overweight	− 0.0025^a^	− 0.082	0.012	− 0.081	0.012
Green → Leisure walking → Overweight	0.0030^a^	− 0.052	0.042	− 0.049	0.045
Walkability → Leisure walking → Overweight	0.0014^a^	− 0.026	0.061	− 0.025	0.062
Safety → Leisure walking → Overweight	0.0006^a^	− 0.070	0.019	− 0.069	0.020
Crowd → Leisure walking → Overweight	0.0009^a^	− 0.053	0.025	− 0.053	0.027

The specific mediating effects in the table refer to the mediating effects for each group.bootstrap method was used to perform 2000 bootsamples for parameter estimation

^a^10% significance level. Sociodemographic characteristics are controlled

### Gender disparities

Table 4 presents the gender-specific influence of the objective versus subjective environment on walking and overweight. Gender disparities exist in terms of pathways between the objective environment and health. For men, lower population density, land use diversity and availability of service facilities are significantly associated with more transport or leisure walking, while mixed land use and lower bus-stop density are positively associated with overweight. However, we observe insignificant impacts of objective environment on female overweight.

**Table 4:** Gender disparities in the standardised direct effects between neighbourhood environment, walking and overweight

	Male group			Female group		
	Transport walking	Leisure walking	Overweight	Transport walking	Leisure walking	Overweight
Individual-level variables						
Age	0.090^**^	0.157^***^	0.047	0.097^***^	0.139^***^	0.196^***^
Marital status	− 0.105^**^	− 0.055	0.175^***^	− 0.020	0.014	− 0.021
Household size	0.030	0.070^*^	0.086^***^	0.022	0.077^***^	0.025
Car ownership	− 0.079^***^	0.039	− 0.021	0.009	− 0.013	− 0.005
Homeownership	0.058^*^	0.008	0.006	0.018	0.077^**^	0.048^*^
Objective environment						
Density	− 0.118^**^	− 0.082^**^	0.017	0.005	0.070^*^	− 0.023
Diversity	0.068^**^	0.060^**^	0.052^*^	− 0.031	-0.002	0.033
Design	0.072	− 0.031	0.019	− 0.015	− 0.073	− 0.051
Access	0.177^***^	0.174^***^	− 0.034	0.027	0.032	0.021
Transit	− 0.007	− 0.055^*^	− 0.083^**^	− 0.049^*^	− 0.134^***^	0.029
Green	0.064	0.123^***^	− 0.011	0.115^***^	0.103^***^	0.006
Subjective environment						
Convenience	− 0.056^*^	0.023	0.271^***^	0.02	0.012	0.141^***^
Walkability	0.027	0.061^**^	0.015	− 0.003	0.054^*^	0.009
Aesthetics	0.005	0.035	− 0.04	− 0.039	− 0.012	− 0.025
Safety	0.071^**^	0.027	− 0.032	0.039	0.026	− 0.004
Crowd	0.004	− 0.028	0.004	0.053	0.086^***^	− 0.022
Walking behaviour						
Transport walking			0.022			0.043^*^
Leisure walking			0.018			− 0.008

^*^p < 0.1

^**^p < 0.05

^***^p < 0.01

The associations between the subjective environment and different patterns of walking are different among male and female respondents. Men are more concerned about transport walking in safety, while women see neighbourhoods with higher community walkability and perceived lively as more pleasant for leisure walking. However, we do not observe significant differences between men and women with respect to the overall influence of subjective environment on overweight.

Significant associations of leisure walking with overweight are observed among female adults. The positive relationship between leisure time activity and weight loss is thought to be achieved with a certain intensity and duration of physical activity. Moreover, it is harder for women to achieve physical activity levels sufficient to control their weight. The average leisure walking time among the women in our survey was about two hours per week, which is not intense enough to achieve the energy expenditure required for weight loss [10, 42, 64].

## Discussion

### The objective and subjective environment, walking behaviour and overweight

This study is based on a localised assessment of the respondents’ residential environment for assessing interactions between the built environment, purpose-specific walking and health outcomes. Our findings suggest that higher accessibility to various service facilities and visible greenery contribute to moderate physical activity, i.e., walking in this study. This affirms that walking behaviour is also strongly affected by eye-level street greenery [38, 53]. Therefore, neighbourhood design with proper service facilities and greenery coverage is conducive to more frequent and long-time walking behaviour. Furthermore, subjective environmental measures, particularly walkability, safety and crowdedness, also influence walking behaviour in Chinese adults, which is consistent with some empirical studies in Europe [13]. Residents in walkable and safe environments may have better social interactions with neighbours, which can positively influence their physical activity.

We argue that objective land use diversity is an important variable in terms of its association with overweight. However, in contrast to previous studies reporting a positive effect of mixed land use on increased non-motorised transportation and reduced risks of overweight based on low-density urban settings in the West [14], the empirical evidence in the Chinese city of Wuhan demonstrates a correlation of increased mixed use with increased risks of overweight. We further argue that the perceived convenience of facilities in the neighbourhood is associated with increased odds of being overweight. This may because that the mixed-use areas or convenient access to facilities expose individuals to a wider variety of food choices, including potentially unhealthy options.

Walking is an easy way for most adults to incorporate more physical activity into their daily routines. Although some studies have noted that increased volumes of any walking may provide health benefits as compared with little or no walking at all, our research reveals the direct and indirect impact of leisure walking on overweight. This may because our survey's low threshold for walking activity (10 continuous minutes) might capture predominantly low-intensity physical activities, which are less likely to contribute significantly to weight control. This is supported by findings from randomized controlled trial in the U.S., which found that a longer continuous walking bout is more effective for hip circumference control than several shorter ones [56]. Additionally, cultural and socio-economic factors could play a role. For example, Turner et al. [ 63] found that the energy expenditure from leisure walking might be offset by increased caloric intake, leading to weight maintenance or gain. This aligns with observations in China's night-time economy, where leisurely strolls often coincide with exposure to high-calorie foods [5]. Furthermore, gender differences in physical activity levels, as noted by the U.S. Department of Health and Human Services (2017) [65], and findings from Petersen et al. [46] that changes in leisure time physical activities are unrelated to subsequent weight change in women, support the complexity of this relationship [37, 74, 77].

### Gender-specific observations

The objective environment has more significant impacts on men’s health outcomes than on women’s. Previous studies have widely examined the effect of the neighbourhood environment on physical health while ignoring gender disparities [77]. We found that overweight was more prevalent among men than women in Wuhan. Men are more at risk of being overweight when they live in a neighbourhood with higher levels of mixed land use, while women are more likely to be overweight if living in a neighbourhood with more perceived convenience. This probably denotes that men and women value the built environment differently, a difference that in turn affects their health-seeking behaviour such as walking or exercise and use of health services [66].

The subjective environment affects men’s transport walking but not women’s leisure walking. Specifically, men are more concerned about perceptions of convenience and safety during transport walking, while women are more concerned about perceptions of walkability and population crowdedness during leisure walking. This gender difference may be attributed to the gender distribution into occupational or social roles [25]. Transport walking is not vigorous enough for men to reduce their risks of being overweight. Women tend to walk more outside work and take part in leisure activities. In our study, women reported more walking time than men and more leisure walking than transport walking. The stronger protective associations of leisure walking with obesity for women suggest that women tend to have more night-time physical activities and nightlife eating, which counterbalances the total energy expenditure and increases their susceptibility to obesity.

### Policy implications

Our results suggest that the six-dimensional objective environment, particularly the availability of service facilities and greenery coverage, influences people’s walking behaviour. Policies and interventions that improve neighbourhood facilities and amenities, such as establishing more healthcare facilities, wet markets and walkways, and maintaining green spaces, may contribute to increasing levels of physical activity. Furthermore, walking behaviour is heavily affected by perceptions of walkability and safety in our study. Residents express concerns about the safety of walking and walkability in their neighbourhood. Pedestrian precincts separating pedestrians from vehicle activities should be created, and public spaces that meet the physical and mental needs of residents should be designed. Local governments should implement policy initiatives such as safe driving campaigns that protect walkers from distracted drivers and new sustainable road infrastructure projects that enhance active mobility and safety.

Considering our finding that leisure walking increases the risk of overweight, strategies to promote physical activity should be coupled with efforts to influence the food environment. Research on healthy food accessibility and “food deserts” demonstrates the potential of intervening in the built environment to support a healthy diet [30]. While mixed land use may promote walking, planners should also consider how to balance the convenience of unhealthy food choices in these environments. One possible approach could be to encourage healthier food retailers in these neighbourhoods, or to implement urban design measures that promote healthier food choices. International practices to address similar issues include zoning by-laws to restrict fast-food outlets in certain areas, intervening in the promotion and display of corner stores, and strengthening healthy retailer skills and collaboration in countries with serious obesity problems, such as the United States and Canada, and in countries such as East Asia, where obesity is on the rise [3, 39, 40, 45].

## Conclusions

This study examines whether and to what extent the objective versus subjective environment influences purpose-specific walking behaviour and overweight in Wuhan, China. The “5D + Greenery” framework is employed to measure the objective environment, defined by population density, land use diversity, road network design, destination accessibility, distance to transit and greenery, by integrating spatial big data and deep learning. The SEM results indicate that the direct effects of objective measures of land use diversity and subjective measures of convenience on overweight are statistically significant. The mediation analysis demonstrates that leisure walking is an important covariate between the neighbourhood environment and overweight. Furthermore, gender-specific disparities are observed in the relations between the built environment, purpose-specific walking, and health outcomes. Our analysis could have implications for policymakers to determine environmental interventions to promote walking and health benefits.

This study also has some limitations. First, while our study considers certain socioeconomic indicators, these indicators may not fully capture the complexity of the self-selection effect. Future studies could benefit from employing more nuanced modelling approaches to directly address this complex issue Second, although studies have recognised the effectiveness of considering household activity in health outcome assessment, not least for women [41], we admit that our gender-specific observations are vulnerable to some bias due to the unavailability of household activity in our study. Third, self-reported data for height and weight could introduce some bias into our BMI calculations. However, this approach was chosen in part to respect participant privacy, as the on-site collection of these measurements may have been viewed as invasive or uncomfortable for some individuals. While we strive to ensure the accuracy of our results, we have taken this into consideration when interpreting our findings. Last but not least, in this study, we only consider the mediating mechanism of purpose-specific walking behaviour on the built environment/health relationship. Future research could expand on other elements, such as sedentary time, household activity, and other exercises that would also affect health outcomes.

## Data Availability

The data and materials in this manuscript are available upon request.

## Footnotes

1 *Jiedao* is the basic administrative unit of Chinese cities. Each *jiedao* is under the administration of a district.
